# Fibrin glue as a sealant in stentless laparoscopic pyeloplasty: A randomised controlled trial

**DOI:** 10.1080/2090598X.2019.1611990

**Published:** 2019-05-09

**Authors:** Ahmed Farouk, Ahmed Tawfick, Mahmoud Reda, Ahmed M. Saafan, Waleed Mousa, Ahmed M. Tawfeek, Hassan Shaker

**Affiliations:** Department of Urology, Ain Shams University Hospitals, Cairo, Egypt

**Keywords:** Laparoscopic pyeloplasty, fibrin glue, PUJ obstruction, stentless pyeloplasty

## Abstract

**Objective**: To evaluate the value of adding fibrin glue, as a sealant material, to the anastomotic line during stentless laparoscopic pyeloplasty (LPP).

**Patients and methods**: In all, 92 patients with pelvi-ureteric junction obstruction (PUJO), scheduled for LPP, were randomised into two groups (46 in each group). Group A, underwent transperitoneal stentless LLP sealed with fibrin glue, whilst Group B underwent the same procedure without fibrin glue.

**Results**: Both groups were similar for patient demographics and presentation. Despite that, we found a significant statistical difference between the groups for operative time and blood loss. The total number of patients that had a urinary leak was 10 and 24 patients, in groups A and B respectively (*P* = 0.002). A prolonged leak lasting for >5 days, which stopped spontaneously occurred in three patients (7.14%) in Group A and six (14.3%) in Group B (*P* = 0.265). A persistent 14-day leak that needed intervention developed in two patients (4.3%) in Group A and five (10.9%) in Group B (*P* = 0.434). One patient in Group B developed urinoma 1 week after discharge, and another patient in the same group developed deep venous thrombosis. There was no significant difference between the groups for postoperative complications in the early 3-month period. The success rate was 39 (92.86%) and 36 patients (85.7%), in groups A and B respectively (*P* = 0.265).

**Conclusion**: Adding fibrin glue to seal the anastomosis decreased urinary leakage but did not have a significant impact on outcomes.

**Abbreviations**: CONSORT: Consolidated Standards of Reporting Trials; DTPA: diethylene-triamine-penta-acetic acid; LPP: laparoscopic pyeloplasty; PUJO: PUJ obstruction; T½: clearance halftime (renogram)

## Introduction

PUJ obstruction (PUJO) occurs in nearly one in 500 to one in 1250 live births. The Anderson-Hynes dismembered pyeloplasty is the ‘gold standard’ for treating PUJO, with the success rate estimated to be as high as 90% [1].

Over the past two decades, laparoscopic pyeloplasty (LPP) has been developing as an alternative procedure and is becoming more or less a standard practice. LPP aims to combine the same excellent results of open pyeloplasty with the advantage of being minimally invasive [2].

The merits of intubated vs non-intubated repair of PUJO have been continuously debated. Many surgeons recommend stenting to prevent urine leakage and to maintain the anastomosis patent, as postoperatively oedema at the anastomotic site may lead to occlusion of the lumen [3]. However, indwelling ureteric stents can cause problems and significant adverse effects, such as discomfort, infection, migration, and encrustation, which can lead to significant morbidity [4]. Additionally, removing them requires an additional procedure and exposure to anaesthesia.

Theoretically, stents can act as foreign bodies causing compromised vascularity and fibrosis at the anastomotic site. Stentless pyeloplasty reduces the risk of infection, avoids the risk of the patient developing stent syndrome and the need for cystoscopic removal [5]. Therefore, there is trend towards stentless LPP, especially when watertight closure can be achieved [6,7].

As there is prolonged leakage and hospital stay in stentless LPP, especially in the paediatric age group [8–10], it is plausible, at least theoretically, that adding fibrin glue on the suture line as a sealant material could be used to decrease urinary leakage and promote healing [11]. Thus, in the present study, we aimed to assess the value and effectiveness of adding fibrin glue, as a sealant material, to the anastomotic suture line during stentless LPP.

## Patients and methods

The primary endpoint of this study was to evaluate urinary leakage, as it is the main disadvantage of stentless pyeloplasty. The secondary endpoints were complications and early outcomes of both groups.

Between March 2013 and June 2016, 187 patients presented to the outpatient urology clinic of the university hospital. They were diagnosed with PUJO and were scheduled for LPP. In all, 92 of those patients underwent transperitoneal dismembered LPP based on the following inclusion and exclusion criteria:

Inclusion criteria:

- Significant loin pain
- Significant pelvi-calyceal dilatation (Grade >II).
- Split renal function lower by 10% as compared to the contralateral normal side.
- Clearance halftime (T½; renogram) >20 min in diuretic renogram.

Exclusion criteria:

- Non-secreting kidney with split function of <15%.
- Previous renal surgery.
- Pyonephrosis.
- Children aged <2 years.
- Bleeding tendency.

Sample size was calculated using the STATA program (Stata Statistical Software: release 12; StataCorp., College Station, TX, USA), setting the type-1 error (α) at 0.05 and the power (1-β) at 0.8. Reviewing the literature we found that there were no similar previous studies. Therefore, we conducted a pilot study, which revealed that one out of five cases in the fibrin glue group compared to three out of six cases in no fibrin glue group had urinary leakage. Calculation according to these values produced a minimal sample size of 39 cases per group, assuming a 10% drop-out rate; the required sample was therefore 43 cases per group. We included 46 cases to achieve better statistical power.

After obtaining informed consent forms from the patients and explaining to them the investigational nature of the study, we divided them into two groups by simple 1:1 randomisation. Each group consisted of 46 patients. In Group A, the anastomotic suture line was sealed with fibrin glue, whilst in Group B no fibrin glue was used. The study was approved by the Faculty of Medicine’s Ethics Committee.

**Figure 1.**
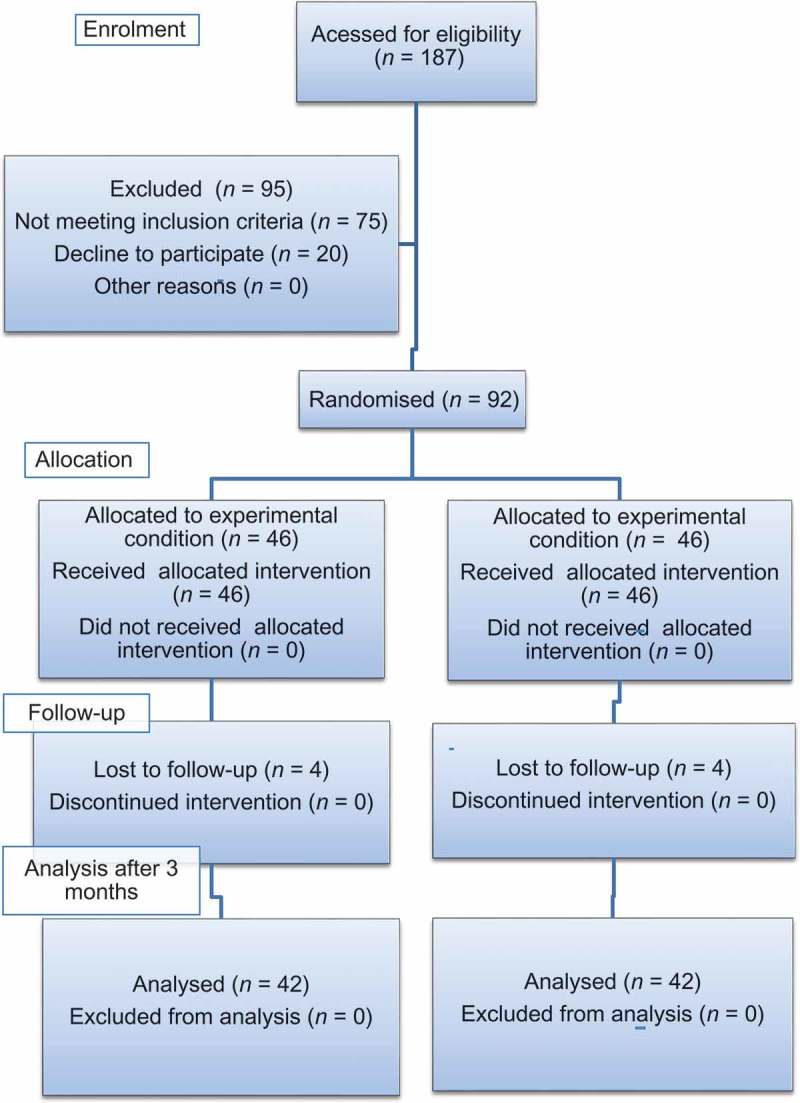
CONSORT flow chart of the study.

The preoperative assessment of patients included: history and physical examination, pelvi-abdominal ultrasonography, IVU with delayed film, (F-15) diuretic renogram (diethylene-triamine-penta-acetic acid [DTPA]) with Lasix, and urine analysis and culture. The study design and work flow are summarised in a Consolidated Standards of Reporting Trials (CONSORT) flow chart (Figure 1).

All patients underwent the procedure under general anaesthesia. The patients were placed in a 45–60° lateral decubitus position. Transperitoneal laparoscopic access was obtained after kocherisation of the colon; the renal pelvis and upper ureter were identified and dissected. After complete exposure of the renal pelvis, excision of the stenotic segment of the PUJ and reduction of the renal pelvis was done. The ureter was spatulated posteriorly for 2–3 cm. Suturing of the PUJ was then completed by two separate continuous sutures of 4-0 polyglycolic acid (Vicryl®; Ethicon Inc., Somerville, NJ, USA), at both sides of the apex of ureteric spatulation to the corresponding most dependent part of the reduced pelvis. Both ‘lips’ of the spatulated ureter were anastomosed to the renal pelvis, starting with the posterior lip first, in a running fashion. The remaining parts of the renal pelvis were sutured together continuously.

In Group A, 5–8 mL semi-solid fibrin glue was prepared and applied by introducing an open-tip 16-F catheter through the 10-mL port. The prepared fibrin glue was then loaded in to a wide-nozzled 60-mL syringe and the glue then injected through the catheter to the anastomotic suture line. In Group B, no fibrin glue was injected.

In both groups, a 16-F tube drain was inserted from one of the ports, under laparoscopic guidance, and it was removed when the amount drained was <50 mL in 24 h.

The following data were recorded: operative time from applying the first port until applying the tube drain, intraoperative complications, postoperative pain (determined using a visual analogue scale, range 0–10), incidence of UTI, amount and duration of urinary leakage, and hospital stay. At the 3-month follow-up the patients were assessed by IVU and DTPA diuretic renogram.

### Preparation of fibrin glue from autologous blood

The fibrin glue was prepared using two constituents, which were stored separately until use. The first component was prepared as follows: 100 mL blood from the patient was withdrawn on sodium citrate 10%. This sample was centrifuged for 8 min at 1923 ***g***. Ethanol 100% was mixed with part of the removed plasma in a 1:7 ratio. This mixture was cooled to –18 °C for 20 min and then re-centrifuged. The precipitate of this centrifugation being fibrinogen pellets. By incubating the rest of the plasma with the fibrinogen pellets at 37 °C for 15 min, the fibrinogen pellets dissolve to give the first component. The second component was prepared by mixing thrombin to 9.2 mL calcium chloride solution (40 mmol/L). This mixture was saved at 37 °C until usage. During the surgery, both components were combined to give a gelatinous substance.

### Statistical analysis

The collected data were revised, coded, tabulated and analysed using the Statistical Package for the Social Sciences (SPSS®), version 20.0 (SPSS Inc., IBM Corp., Armonk, NY, USA). Quantitative variables are expressed as mean (SD) or as median (range) in cases of non-parametric variables. Qualitative variables are expressed as frequencies and percentage. The Student’s *t*-test and Mann–Whitney tests were used to compare continuous variables of the two study groups when appropriate. A paired *t*-test was used to compare a continuous variable of the same group. Chi-squared and Fisher’s exact tests were used to compare qualitative variables of the two study groups when appropriate. A *P* < 0.05 was considered statistically significant.

### Ethical considerations

The present study was approved by the Faculty of Medicine’s Ethics Committee. Written informed consent was obtained from all study subjects. The study was not funded by external sources and there was no conflict of interest.

## Results

There were no statistically significant differences between the two study groups for patients’ demographics. (Table 1)

**Table 1.** Patients’ demographic and preoperative data.

Variable	Group A (fibrin)	Group B (no fibrin)	*P*
Age, years, mean (SD; range)	20.96 (13.77; 6.00–45.00)	19.72 (12.18; 3.50–48.00)	0.649
Gender, *n* (%) Male Female	34 (73.9) 12 (26.1)	28 (60.9) 18 (39.1)	0.182
Affected side, *n* (%) Right Left	20 (43.5) 26 (56.5)	15 (32.6) 31 (67.4)	0.283
Significant loin pain, *n* (%)	36 (78.3)	38 (82.6)	0.599
Non-secreting kidney	10 (21.7)	6 (13.0)	0.271
Pelvi-calyceal dilatation at IVU	34 (73.9)	36 (78.3)	0.625
T½, min, mean (SD; range)	27.30 (8.65: 17.00–55.00)	30.72 (12.90; 17.75–80.00)	0.140
Split renal function, %, mean (SD; range)	34.96 (7.80; 19.00–50.00)	31.36 (7.62; 17.56–42.04)	0.028

No statistical significant difference with regards to patient demographics and patients presentation of both groups, except for split renal function

There was no significant difference in presentation of both groups, except for split renal function, where the fibrin group (Group A) had a higher mean compared to the no fibrin group (Group B; Table 1).

For the intraoperative data, there was a significant difference between the fibrin and no fibrin group for operative time, which was higher in the fibrin group, at a mean (SD) of 186.4 (49.5) vs 164.8 (46.46) min. Additionally, there was a significant difference between both groups in blood loss, where the no fibrin group had greater blood loss. No statistically significant difference was found between both groups for visceral or vascular injury, and there were no conversions to open surgery in either group (Table 2).

**Table 2.** Operative, early postoperative, and 3-month postoperative data.

Variable	Group A (fibrin)	Group B (no fibrin)	*P*
**Operative data**			
Operative time, min, mean (SD; range)	186.39 (49.50; 120.0–311)	164.78 (46.46; 120.0–300)	**0.034**
Blood loss, mL, mean (SD; range)	31.26 (25.00; 10.0–100)	56.80 (63.14; 10.0–250)	**0.012**
Vascular injury, *n* (%)	0	1 (2.2)	1.0
**Early postoperative data**			
VAS pain score, mean (SD; range)	5.22 (1.63; 3.0–9)	5.04 (1.55; 2.0–8)	0.601
Urine leakage, mL/day, mean (SD; range)	299.78 (192.93; 50.0–700)	325.54 (315.91; 30.0–1300)	0.638
Duration of urinary leakage, days, mean (SD; range)	4.61 (2.78; 1.0–14)	4.83 (3.84; 1.0–14)	0.756
Drain removal, days, mean (SD; range)	6.61 (2.78; 3.0–16)	6.98 (4.23; 3.0–19)	0.622
Hospital stay, days, mean (SD; range)	5.70 (2.80; 4.0–16)	6.98 (4.23; 3.0–19)	0.09
**3-month postoperative complications, *n* (%)**			
Haematuria	2 (4.3)	2 (4.3)	1.0
Pyelonephritis	8 (17.4)	14 (30.4)	0.143
Pyuria	8 (17.4)	9 (19.6)	0.788
**3-month postoperative data**			
T½, min, mean (SD; range)	12.33 (6.28; 4.00–34.00)	14.93 (8.88; 5.00–45.00)	0.115
Split renal function, %, mean (SD; range)	39.22 (9.70; 18.00–53.97)	35.65 (10.82; 12.00–53.97)	0.126
Deterioration, *n* (%) No Yes	39 (92.86) 3 (7.14)	36 (85.7) 6 (14.3)	0.265
Lost to follow-up, *n* (%) No Yes	42 (91.3) 4 (8.7)	42 (91.3) 4 (8.7)	1.0

No statistically significant difference for intraoperative data and early postoperative complications. At the 3-month follow-up, there was significant an improvement in the T½ and split renal function for both groups but no difference between the two groups.

**Table 3.** Postoperative complications summarised according to Clavien classifications.

Complication grade	Group A (fibrin), *n*	Group B (no fibrin), *n*
I	3 prolonged leakage 2 haematuria	6 prolonged leakage 2 haematuria
II	8 pyuria 8 pyelonephritis	9 pyuria 14 pyelonephritis 1 (deep venous thrombosis)
III	2(urinary leak) 3 (endopyelotomy)	5(urinary leak) 1(urinoma) 6 (endopyelotomy)
IIIa	2	2
IIIb	3	10

In the early postoperative period, no significant difference was found for postoperative pain, amount and duration of leakage, time to drain removal, and hospital stay. Nevertheless, the number of patients that had prolonged urinary leakage was more than double in the no fibrin group vs the fibrin group (10 and 24 patients, respectively), which was highly statistically significantly different (*P* = 0.002). From those, urinary leakage occurred in five patients in Group A and 12 patients in Group B in the immediate postoperative period, but did not last for >5 days. Prolonged leakage continued for of >5 days in three patients (7.14%) in Group A and six (14.3%) in Group B, which stopped spontaneously before the end of 14 days, a statistically insignificant difference (*P* = 0.265). Additionally, two (4.3%) and five patients (10.9%), in groups A and B respectively, developed a persistent leak for 14 days. This was again statistically insignificant (*P* = 0.434). All patients with persistent leakage were managed by inserting a JJ stent, except in one child aged 3.5 years in Group B, who was managed by nephrostomy due to failure of applying the JJ stent. One patient in Group B, a 9-year-old child, developed urinoma a week after he was discharged. He was managed by nephrostomy and pigtail catheter drainage. Finally, one patient in Group B developed a deep venous thrombosis (Table 2).

There were no significant differences between the fibrin and no fibrin groups for other early postoperative complications (Table 3).

At the 3-month follow-up, there was significant improvement in the T½ and split renal function in both groups. In Group A, 39 patients (92.86%) had a downgrading of pelvi-calyceal dilatation and an improved mean T½ and mean split renal function, whilst three patients (7.14%) had deterioration of pelvi-calyceal dilatation with prolonged T½ and a worsening of split renal function. These patients were managed with endopyelotomy. We had four patients who missed their follow-up in this group. In Group B, 36 patients (85.7%) had a downgrading of pelvi-calyceal dilatation and an improved mean T½ and mean split renal function, whilst six patients (14.3%) had deterioration of pelvi-calyceal dilatation with prolonged T½ and deterioration of split renal function. They were also managed with endopyelotomy. Four patients missed their follow-up in this group (Table 2). There were no statistically significant differences between the groups with for postoperative IVU, mean split renal function, and mean T½ (Table 2).

## Discussion

Although Anderson and Hynes [12] first described dismembered pyeloplasty for the treatment of an obstructed retrocaval ureter in 1949, since then open pyeloplasty has been viewed as the ‘gold standard’ surgery for PUJO, with a success rate of >90% [13].

The treatment of PUJO changed considerably in 1993, when Schuessler et al. [14] first introduced LPP. Shortly afterwards, LPP emerged as an alternative first-line option in the management of PUJO, with a success rate that parallels that of the open approach [15].

Anderson and Hynes [12] own comment on their own technique, stating: ‘Splinting of any anastomosis is not only unnecessary but it is against all the principles of plastic procedure, as it leads to infection and fibrosis at the line of suture and subsequent stricture. The line of anastomosis should be wide enough and so fashioned as to render any subsequent contraction’. So JJ stenting is an important aide to LPP but is associated with significant morbidity [16]. Thus, stentless repair after LPP was strongly advocated over time.

Urinary leakage, urinoma formation, and a longer hospital stay, are the main drawbacks of stentless repairs in either laparoscopic or open surgery [8–10,16].

Fibrin sealant is a mixture of coagulation factors (thrombin and highly concentrated fibrinogen), with haemostatic and adhesive properties. It has been traditionally used for three major reasons in urological surgery. First, as a haemostatic agent, in open and laparoscopic partial nephrectomy, percutaneous nephrolithotomy, management of splenic injury, haemophilia and other coagulopathy, circumcision, and haemorrhagic cystitis. Second, as a urinary tract sealant, such as in procedures like LPP and open pyeloplasty, ureteric anastomoses, urethral reconstruction, simple retropubic prostatectomy, radical retropubic prostatectomy, vasovasostomy and vasoepididymostomy, bladder injury, and percutaneous nephrolithotomy tract closure. Lastly, as a tissue adhesive and healing promotor in Fournier’s gangrene, fistula closure, skin grafting, orchidopexy, penile chordee, and complex urethroplasty [11]. Therefore, at least theoretically, issues such as urinary leakage, urinoma formation and hospital stay can be addressed or decreased by adding fibrin glue to the anastomotic suture line.

To our knowledge, there is paucity of published literature on fibrin glue usage for sealing the anastomotic suture line in stentless LPP in humans. In addition to that, there are few reports about applying fibrin glue for ureteric repairs and pyeloplasty, even though Kram et al. [17] in 1989 suggested that fibrin glue could be used to decrease the leakage of urine in cases of ureteric injuries.

In 1997, Eden et al. [18] assessed the results of fibrin-glued dismembered pyeloplasty in patients with PUJO by applying a JJ stent. They reported that the procedure was completed in eight patients and that one patient was converted to open surgery due to a short fibrotic ureter. The median (range) postoperative hospitalisation was 2 (2–4) nights. In the aforementioned study, the urine leakage was minimal and the drain was removed on the second day postoperatively. The success rate was a 100%. Only one patient developed a renal pelvic calculus, which was treated by JJ stent and shockwave lithotripsy.

In 2016, we reported on the effectiveness of fibrin glue as a sealant in stentless LPP and compared it to stented LPP. Although the mean values of urinary leakage and hospital stay were almost equal in both groups, three patients in the stentless group had prolonged urinary leakage and therefore had longer hospital stays (>7 days) than the patients in the stented group [19].

In the present study, with regards to the primary endpoint, the total number of patients that had prolonged urinary leakage, whether in the immediate postoperative period or persisting for a longer time, was more than double in Group B (no fibrin) compared to Group A. Although the difference did not attain statistical significance, it was nevertheless clinically important (*P* = 0.06). When we broke down these patients in to three subgroups according to timing of leakage and the need for intervention, the difference did not reach statistical significance, although it was higher in Group B in each of the subgroups. The number of the patients that needed stenting in Group B was again more than double those in Group A. Again, this did not reach statistical significance. This is probably due to the small number of patients in the subgroups. Regarding the secondary endpoints, regardless of whether fibrin glue was used or not, stentless LPP achieved a high success rate. Again, this was marginally better in the fibrin glue group [39 patients (92.86%) in Group A and 36 patients (85.7%) in Group B] and again the difference was statistically insignificant.

Although adding fibrin glue at the anastomotic suture line is merely one simple additional step in LPP, there are few operative points that are noteworthy. The operative time was significantly longer in the fibrin group, a mere few minutes that were needed to constitute and apply the fibrin. On the other hand, LPP is a bloodless operation; blood loss was significantly greater in the no fibrin group due to gonadal injury and possibly due to the lack of the haemostatic effect of fibrin glue. Again, this was not more than a clinically insignificant few millilitres.

We encountered several limitations in our present study, namely, the small sample size, the short-term follow-up, and the inclusion of both paediatric and adult age groups within the same study.

## Conclusion

Adding fibrin glue at the anastomosis suture line can decrease urinary leakage but does not improve outcome significantly. Fibrin glue is recommended if the surgeon prefers a stentless LPP.

## References

[1] Hopf HL, Bahler CD, Sundaram CP. Long-term outcomes of robot-assisted laparoscopic pyeloplasty for ureteropelvic junction obstruction. Urology. 2016;90:106–110. 26801810 10.1016/j.urology.2015.12.050

[2] Ekin RG, Celik O, Ilbey YO. An up-to-date overview of minimally invasive treatment methods in ureteropelvic junction obstruction. Cent European J Urol. 2015;68:245–251. 10.5173/ceju.2015.543 PMC4526614 26251754

[3] Meisheri IV, Kamat TA and Maheshwari M. Pelviureteric junction obstruction - stented versus unstented pyeloplasty. J Indian Assoc Pediatr Surg. 2004;9:184–188.

[4] Milicevic S, Bijelic R, Jakovljevic B. Encrustation of the ureteral double j stent in patients with a solitary functional kidney - a case report. Med Arch. 2015;69:265–268. 26543316 10.5455/medarh.2015.69.265-268 PMC4610658

[5] Nguyen DH, Aliabadi H, Ercole CJ, et al Nonintubated Anderson-Hynes repair of ureteropelvic junction, observation in 60 patients. J Urol. 1989;142:704–706 . 2671411 10.1016/s0022-5347(17)38859-6

[6] Shalhav AL, Mikhail AA and Orvieto MA, et al Adult stent less laparoscopic pyeloplasty. JSLS. 2007 ; 11:8–13. 17651549 PMC3015796

[7] Bilen CY, Bayazit Y, Güdeloðlu A, et al Laparoscopic pyeloplasty in adults: stented versus stentless. J Endourol. 2011;25:645–50. 21381956 10.1089/end.2010.0401

[8] Smith KE, Holmes N, Lieb JI, et al Stented versus nonstented pediatric pyeloplasty: a modern series and review of the literature. J Urol. 2002;168:1127–30. 12187251 10.1016/S0022-5347(05)64607-1

[9] Kumar V, Mandhani A. Laparoscopic stentless pyeloplasty: An early experience. Indian J Urol. 2010;26:50–55. 20535285 10.4103/0970-1591.60444 PMC2878438

[10] Elmalik K, Chowdhury MM, Capps SN. Ureteric stents in pyeloplasty: a help or hindrance? J Pediatr Urol. 2008;4:275–279. 18644529 10.1016/j.jpurol.2008.01.205

[11] Evans LA, Morey AF. Hemostatic agents and tissue glues in urologic injuries and wound healing. Urol Clin North Am 2006;33:1–12. 16488275 10.1016/j.ucl.2005.10.004

[12] Anderson JC, Hynes W. Retrocaval ureter; a case diagnosed pre-operatively and treated successfully by a plastic operation. Br J Urol. 1949; 21: 209–14. 18148283 10.1111/j.1464-410x.1949.tb10773.x

[13] O’Reilly PH, Brooman PJ, Mak S, et al The long-term results of Anderson-Hynes pyeloplasty. BJU Int 2001;87: 287–289. 11251517 10.1046/j.1464-410x.2001.00108.x

[14] Schuessler WW, Grune MT, Tecuanhuey LV, et al Laparoscopic dismembered pyeloplasty. J Urol. 1993;150:1795–9. 8230507 10.1016/s0022-5347(17)35898-6

[15] Nishi M, Tsuchida M, Ikeda M, et al Laparoscopic pyeloplasty for secondary ureteropelvic junction obstruction: long-term results. Int J Urol. 2015;22:368–371. 25599801 10.1111/iju.12686

[16] Woo HH, Farnsworth RH. Dismembered pyeloplasty in infants under the age of 12 months. Br J Urol. 1996;77:449–451. 8814855 10.1046/j.1464-410x.1996.89723.x

[17] Kram HB, Ocampo HP, Yamaguchi MP, et al Fibrin glue in renal and ureteral trauma. Urology. 1989;33:215–218. 2465646 10.1016/0090-4295(89)90395-6

[18] Eden CG, Sultana SR, Murray KH, et al Extraperitoneal laparoscopic dismembered fibrin-glued pyeloplasty: medium-term results. Br J Urol. 1997;80:382–389. 9313653 10.1046/j.1464-410x.1997.00367.x

[19] Farouk A, Tawfick A, Kotb M, et al Use of fibrin glue as a sealant at the anastomotic line in laparoscopic pyeloplasty: A randomized controlled trial. Arab J Urol 2016;14:292–298. 27900220 10.1016/j.aju.2016.08.002 PMC5122749

